# Effects of blood flow restriction training on bone metabolism: a systematic review and meta-analysis

**DOI:** 10.3389/fphys.2023.1212927

**Published:** 2023-08-09

**Authors:** Xiaolin Wang, Yifei Wang, Xuezhen Yang, Nasnoor Juzaily Bin Mohd Nasiruddin, Delong Dong, Shamsulariffin Bin Samsudin, Xin-Min Qin

**Affiliations:** ^1^ Department of Sport Studies, Faculty of Educational Studies, University Putra Malaysia, Serdang, Selangor, Malaysia; ^2^ Department of Physical Education, Ludong University, Yantai, China; ^3^ School of Nursing, Shandong First Medical University, Jinan, China; ^4^ Department of Smart Health Science and Technology Convergence (Sport Science), Department of Sport Science, Kangwon National University, Chuncheon, Republic of Korea

**Keywords:** blood flow restriction training, occlusion training, osteogenesis, ischaemia, therapeutic occlusion

## Abstract

**Introduction:** The efficacy of low-intensity blood flow restriction (LI-BFR) training programs in bone metabolism remains unclear compared to low-intensity (LI) training and high-intensity (HI) training. The aim of this review was to quantitatively identify the effects of LI-BFR training on changes in bone formation markers (i.e., bone-specific alkaline phosphatase, BALP), bone resorption (i.e., C-terminal telopeptide of type I collagen, CTX) and bone mineral density (BMD) compared with conventional resistance training programmes. Additionally, the effectiveness of walking with and without BFR was assessed.

**Methods:** PubMed, Scopus, SPORTDiscus, Web of Science and Google Scholar databases were searched for articles based on eligibility criteria. Review Manager Version 5.4 was used for Meta-analysis. Physiotherapy Evidence Database (PEDro) was applied to assess the methodological quality of studies.

**Results:** 12 articles were included in the meta-analysis, with a total of 378 participants. Meta-results showed that compared with LI training, LI-BFR training induced greater increments in BALP (young adults: MD = 6.70, *p* < 0.001; old adults: MD = 3.94, *p* = 0.002), slight increments in BMD (young adults: MD = 0.05, *p* < 0.00001; old adults: MD = 0.01, *p* < 0.00001), and greater decrements in CTX (young adults: MD = −0.19, *p* = 0.15; old adults: MD = −0.07, *p* = 0.003). Compared with HI training, LI-BFR training produced smaller increments in BALP (young adults: MD = −6.87, *p* = 0.24; old adults: MD = −0.6, *p* = 0.58), similar increments in BMD (MD = −0.01, *p* = 0.76) and similar decrements in CTX (young adults: MD = 0, *p* = 0.96; old adults: MD = −0.08, *p* = 0.13). Although there were only two studies on walking training intervention, walking training with BFR had a better effect on bone metabolism than training without BFR.

**Discussion:** In conclusion, LI-BFR training induces greater improvements in bone health than LI training, but is less effective than HI training. Therefore, LI-BFR training may be an effective and efficient way to improve bone health for untrained individuals, older adults, or those undergoing musculoskeletal rehabilitation.

**Clinical Trial Registration:** [https://www.crd.york.ac.uk/prospero/], identifier [CRD42023411837].

## Introduction

It is well established that HI training (≥70% of one-repetition maximum, 1RM) is an effective stimulus for the maintenance of bone health in general populations (Maddalozzo and Snow, 2000; Kohrt, 2004; ACSM, 2009; Kitsuda et al., 2021). However, untrained individuals, older adults, or those undergoing musculoskeletal rehabilitation are not physically capable of withstanding high mechanical loads. LI exercise seems to be an optimal option for those populations, but LI exercise rarely improved bone density (Witzke and Snow, 2000; Linero and Choi, 2021). There is evidence suggesting that LI training (20%–30% 1RM) combined with blood flow restriction (BFR) may provide a novel approach to induce adaption in bone (Karabulut et al., 2011; Freitas et al., 2021).

BFR training utilizes pressure cuffs to occlude venous flow yet allow partial arterial inflow on the proximal aspect of a limb during exercise (Scott et al., 2015; Vanwye et al., 2017). This venous occlusion method could elevate intramedullary pressures and interstitial fluids through increased vascular restriction, which proved to be conducive to bone metabolism (Loenneke et al., 2012). Moreover, BFR may affect osteoclast activity by hypoxia and small reductions in pH (McCarthy, 2006), thereby activating factors (e.g., hypoxia-inducible transcription, vascular endothelial growth) important for neovascularization in bone tissue (Araldi and Schipani, 2010). These blood vessels could transport osteoblasts and osteoclasts that are critical for bone remodeling (Lafage-Proust and Roche, 2019). Additionally, hormones (e.g., cortisol, testosterone, insulin-like growth factor-1) play a vital role in modulating bone metabolism by promoting bone formation or inhibiting bone resorption (Hamdy and Appelman-Dijkstra, 2018; Kraemer et al., 2020; Shigehara et al., 2021; Yinghao et al., 2021).

Bone remodeling consists of resorption, reversal, and formation phases, which take approximately 6 months (Agerbaek et al., 1991). Therefore, bone turnover markers, biomarkers reflecting bone formation and bone resorption, are more effective than BMD in assessing bone responses to exercise interventions in a shorter duration (<6 months). Bone formation markers, such as BALP, osteocalcin (OC) and procollagen I intact N-terminal (P1NP), determine osteoblast activity and bone mineralization (Shetty et al., 2016). On the other hand, bone resorption markers, such as CTX, N-terminal telopeptide of type I collagen (NTX), and deoxypyridinoline (DPD), determine osteoclast activity and bone degradation (Shetty et al., 2016).

The conclusion that BFR could improve bone health was originally based on animal experiments (Qin et al., 2003; Stevens et al., 2006; Qin and Lam, 2009). However, there is still considerable debate regarding the comparative effects of LI-BFR training and LI or HI training on human bone health recently. Two investigations by Beekley et al. (2005) and Park et al. (2019) showed that LI-BFR training had a significantly greater increment in BALP than LI training among young men (10.8% *vs*. 0.3%) and elderly women (7.8% *vs*. 1%), while the research by Fakhri et al. (2021) demonstrated that there was no significant difference in BALP increment between LI-BFR and LI training among inactive females. Zaravar et al. (2021) reported that LI-BFR training had a significantly greater increment in BMD than LI training among elderly women (4.4% *vs*. 1.5%), while two other researches by Karabulut et al. (2011) and Kargaran and Amani-Shalamzari (2022) demonstrated that there was no significant difference in BMD increment between LI-BFR and LI training among elderly men and women. Moreover, the research by Fakhri et al. (2021) showed that LI-BFR training and HI training had a similar effect on BALP among inactive females, but Kim et al. (2012) reported that HI training had a significantly greater increment in BALP than LI-BFR training among young men (39.4% *vs*. 4.2%). There is at the present time no consensus on which training method is more beneficial for the improvement of bone health.

There was currently no meta-analysis review summarizing the effects of BFR training on bone metabolism. Thus, the main aim of this meta-analysis was to investigate the effects of BFR training on bone metabolism and provide practical implications for the prevention and treatment of osteoporosis. The objectives of this review were to 1) compare the effectiveness of LI-BFR training with both LI and HI resistance training without BFR 2) compare the effectiveness of aerobic training (e.g., walking); with BFR and without BFR; 3) systematically review relevant studies and provide recommendations regarding safe and effective implementation of BFR training for untrained individuals, old adults, or those undergoing musculoskeletal rehabilitation.

## Methods

### Experimental approach to the problem

This systematic review was conducted according to the guidelines for Systematic Reviews and Meta-analyses provided in the PRISMA statement (Moher et al., 2009) (Prospero registration number: CRD42023411837).

### Eligibility criteria

BFR intervention studies (not acute studies) involving bone outcomes were included in this analysis. Studies were required to compare LI-BFR training with either low-intensity (<50% 1RM) or high-intensity (≥70% 1RM) training without BFR. Only randomized controlled original articles were included in this meta-analysis. Additionally, studies with a lower than four on the Physiotherapy Evidence Database (PEDro) scale were excluded.

### Information sources

Articles published up to March 28, 2023, were located using the electronic databases PubMed, Scopus, SPORTDiscus, Web of Science and Google Scholar. The search strategy was conducted using the Boolean operators AND and OR with the following keywords: “blood flow restriction”, “vascular occlusion”, “KAATSU”, “bone”, “osteogenesis”, “osteoporosis”, “osteopenia”, “training”, “intervention”. An example of a PubMed search: (“blood flow restriction” OR “vascular occlusion” OR “KAATSU”) [MeSH Terms], (“blood flow restriction” OR “vascular occlusion” OR “KAATSU”) AND (“bone” OR “osteogenesis” OR “osteoporosis” OR “osteopenia”) AND (“training” OR “intervention”) [Title/Abstract]. The lead author’s personal libraries and gray literature sources (e.g., conference proceedings) were also examined. The systematic search process was conducted by Y.X. and Q.X. Any disagreement for inclusion or exclusion of a study was resolved by the third author (S.S.).

### Study selection and data collection process

**FIGURE 1:**
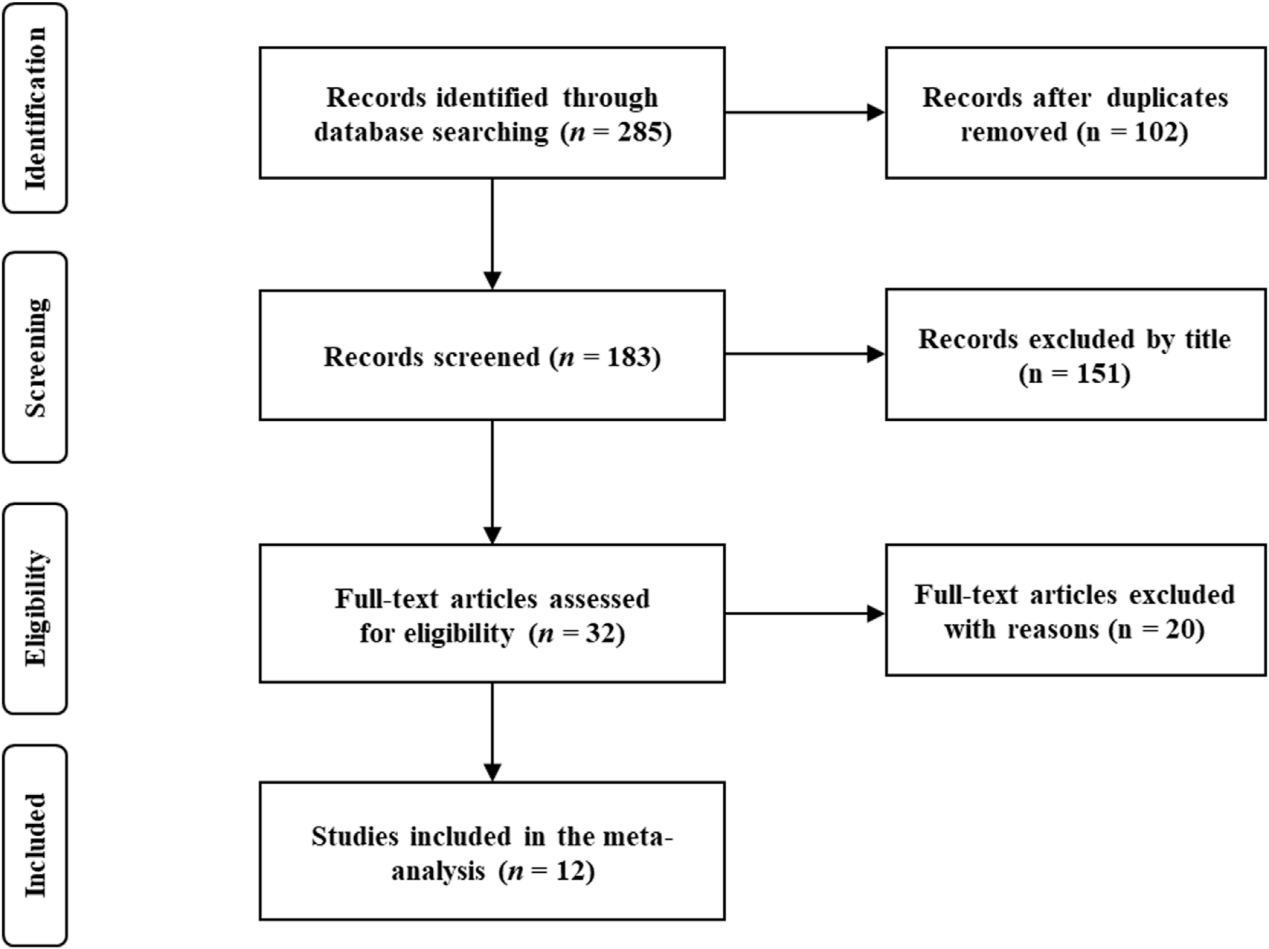
Flow chart.

After excluding duplicate articles, a review of retrieved article titles was conducted. Then, examination of article abstracts and full articles followed (Figure 1). The data extracted from gathered articles were recorded by using Microsoft Excel (Microsoft Corporation, Redmond, WA, United States). Data extraction from the included studies was independently performed by two authors (S.J. and X.M.). Any disagreement in data extraction was resolved by the consensus third author (S.S.).

### Data items

For the current review, data on bone turnover makers (i.e., formation and resorption makers) and bone mineral density (BMD) were extracted. Bone formation markers, such as BALP, OC and P1NP, determine osteoblast activity and bone mineralization (Shetty et al., 2016). On the other hand, bone resorption markers, such as CTX, NTX and DPD, determine osteoclast activity and bone degradation (Shetty et al., 2016).

Extracted data also included the following information: 1) population characteristics: age, sex and health level; 2) interventional characteristics and protocol: mode, frequency, duration, load, and the BFR strategies (i.e., type, cuff pressure and width); 3) main results of the study. The specific characteristics of the included studies were shown in Table 1.

**TABLE 1:** Study characteristic.

Author	Study sample	Protocol & N	Exercise mode	Duration & frequency	BFR type, pressure & width	Outcomes (percentage increase or decrease)
Beekley et al. (2005)	Healthy men (21–28 y)	Walk-CG, 9 Walk-BFR, 9	15-min walk on the treadmill	3 wk; 2 times/day	Continuous BFR; 160–230 mmHg; NG	BALP: Walk-BFR ↑ 10.8%, Walk-CG ↑ 0.3%; IGF-1: Walk-BFR ↑ 3.5%, Walk-CG ↓ 0.2%
Bemben et al. (2022)	Active healthy men (18–35 y)	HI (70% 1RM), 12 LI-BFR (20%1RM), 12 CG (normal daily PA), 8	Upper body: lat pull down, shoulder press, biceps curl, and triceps extension. Lower body: knee flexion and knee extension	12 wk; 2 days/wk	Int-BFR; 120–180 mmHg; 5 cm	BALP: HL ↑ 3.4%, ML ↓ 5.8%, LI-BFR ↑ 0%, CG ↑ 0%; CTX: HL ↓ 12.4%, ML ↑ 0.9%, LI-BFR ↓ 2%, CG ↑ 3%
Fakhri et al. (2021)	Inactive female students (23.84 ± 1.1 y)	LI, 12, LI-BFR, 12, CG, 12	Plyometric training: deep, step, forward and side-to-side jump	4 wk; 3 days/wk	Int-BFR; 160 mmHg; NG	BALP: LI-BFR ↑ 16.2%, LI ↑ 0.4%, CG ↓ 0.2%; CTX: LI-BFR ↓ 4.9%, LI ↓ 2%, CG ↑ 1.2%
Fakhri et al. (2021)	Inactive females (22.96 ± 1.03 y)	HI, 12, LI-BFR, 12, LI, 12, CG, 12	Plyometric training: deep, step, forward and side-to-side jump	4 wk; 3 days/wk	Int-BFR; 160 mmHg; NG	BALP: HL ↓ 0.4%, LI-BFR ↓ 1%, LI ↑ 0.5%, CG ↑ 0.2%; CTX: HL ↓ 10.6%, LI-BFR ↓ 13.2%, LI ↑ 2%, CG ↑ 0.8%
Jack et al. (2023)	Patients (ACL) (24.1 ± 7.2 y)	LI (20% 1RM), 15, LI-BFR (20% 1RM), 17	Leg extension	12 wk; 2 days/wk	NG; 80% AOP; NG	BMD (week 6): LI ↓ 7.6%, LI-BFR ↓ 2.8%; BMD (week 12): LI ↓ 8.2%, LI-BFR ↓ 4.9%
Karabulut et al. (2011)	Healthy elderly men (58.8 ± 0.6 y)	HI (80% 1RM), 13, LI-BFR (20%1RM), 13, CG (normal daily PA), 11	Upper body: latissimus puLI down, shoulder press, and biceps curl. Lower body: leg press and knee extension	6 wk; 3 days/wk	Int-BFR; 120–180 mmHg; 5 cm	BMD: HL ↑ 0%, LI-BFR ↑ 0%, CG ↑ 0.8%; BMC: HL ↓ 0.3%, LI-BFR ↑ 0%, CG ↑ 0%
Kargaran and Amani-Shalamzari (2022)	Elderly women (63.1 ± 2.9 y)	LI, 10, LI-BFR, 10, CG, 10	20-min cognitive-walking training on treadmill	9 wk; 3 days/wk	NG; 50%–80% AOP; 5 cm	BMD: LI ↑ 0%, LI-BFR ↑ 2.1%, CG ↑ 0%
Kim et al. (2012)	Untrained men (18–35 y)	HI (80% 1RM), 10, LI-BFR (20% 1RM), 10, BFR, 10	leg press, knee extension	3 wk; 3 days/wk	Continuous BFR; 120 mmHg; 5 cm	BALP: HL ↑ 39.4%, LI-BFR ↑ 4.2%, BFR ↓ 1.7%; CTX: HL ↑ 1.8%, LI-BFR ↑ 5.7%, BFR ↑ 3.8%
Lambert et al. (2019)	Active adults (23 ± 7 y)	LI (20% 1RM), 7, LI-BFR (20% 1RM), 7	Quadriceps contractions, bilateral leg press, eccentric leg press, hamstring curl, eccentric hamstring curl	12 wk; 2 days/wk	NG; 80% AOP; NG	BMD (week 6): LI ↓ 3.42–13.49%, LI-BFR ↓ 3.55–4.55%; BMD (week 12): LI ↓ 10.35–15.9%, LI-BFR ↓ −1.66‒7.41%
Linero and Choi (2021)	Postmenopausal women (56 ± 1.8 y)	HI (60–80% 1RM), 7, LI (30% 1RM), 6, LI-BFR (30% 1RM), 7, CG, 6	Bilateral leg press, leg extension, dumbbell biceps curl, and triceps extension	12 weeks with a 48-h interval	NG; 140–200 mmHg; 7.5 cm	BMD: HL ↓ 1.1%, LI-BFR ↓ 2.2%, LI ↓ 0.35%, CG ↓ 4.3%; CTX: HL ↑ 21.7%, LI-BFR ↓ 11.5%, LI ↑ 0%, CG ↓ 3.5%
Park et al. (2019)	Elderly women (78.4 ± 6.97 y)	LI-BFR (20% 1RM), 8, LI (20% 1RM), 5, HI (70% 1RM), 8	knee extension, leg curl	12 wk; 3 days/wk	Int-BFR; 120–160 mmHg; 5 cm	BALP: HL ↑ 18.3%, LI-BFR ↑ 7.8, LI ↑ 1%; CTX: HL ↓ 20.5%, LI-BFR ↓ 18.4%, LI ↓ 2.4%
Zaravar et al. (2021)	Elderly women (60–70 y)	LI-BFR,15, LI, 15, CG, 15	Water resistance exercise	8 wk; 3 days/wk	NG; 110–220 mmHg; 5 cm	BMD: LI-BFR ↑ 4.4%, LI ↑ 1.5%, CG ↓ 1.8%

ACL, anterior cruciate ligament; AOP, arterial occlusion pressure; BALP, bone-specific alkaline phosphatase; BMC, bone mineral content; BM, bone mass; BMD, bone mineral density; CTX, C-terminal telopeptide of type Ⅰ collagen; HI, high intensity; IGF-1, insulin-like growth factor 1; Int-BFR, intermittent blood flow restriction; LI, low intensity; LI-BFR, low intensity blood flow restriction; NG, not given; NTX, serum cross-linked N-telopeptide of type Ⅰ collagen; P1NP, procollagen Ⅰ intact N-terminal; PTH, parathyroid hormone.

### Methodological quality of included studies

The Physiotherapy Evidence Database (PEDro) scale (Maher et al., 2003; De Morton, 2009) was used to assess the risk of all included studies. There are 11 items in the PEDro checklist for a total of 10 points (item 1 is not rated). As in a similar previous plyometric training meta-analysis (Stojanović et al., 2017), literature quality was interpreted as “low quality” (≤3 points), “medium quality” (4‒5 points), or “high quality” (6–10 points). The results of the literature evaluation included in this study are shown in Table 2.

**TABLE 2:** Physiotherapy evidence database (PEDro) scale ratings.

References	Items*											Total (from a possible maximal of 10)
	1	2	3	4	5	6	7	8	9	10	11	
Beekley et al. (2005)	1	1	0	1	0	0	1	1	1	1	1	7
Bemben et al. (2022)	1	1	0	0	0	0	1	1	1	1	1	6
Fakhri et al. (2021)	1	1	0	0	0	0	1	1	1	1	1	6
Fakhri et al. (2021)	1	1	0	0	0	0	1	1	1	1	1	6
Kargaran and Amani-Shalamzari (2022)	1	1	0	1	0	0	1	1	1	1	1	7
Karabulut et al. (2011)	1	0	0	1	0	0	1	1	1	1	1	6
Kargaran and Amani-Shalamzari (2022)	1	1	1	1	0	0	1	0	1	1	1	7
Kim et al. (2012)	1	1	0	1	0	0	1	1	1	1	1	7
Lambert et al. (2019)	1	1	0	0	0	0	0	1	1	1	1	5
Linero and Choi (2021)	1	1	0	0	0	0	1	1	1	1	1	6
Park et al. (2019)	1	0	0	0	0	0	1	1	1	1	1	5
Zaravar et al. (2021)	1	1	0	0	0	0	1	1	1	1	1	6
Median score = 6												

* a detailed explanation for each PEDro scale item can be accessed at https://pedro.org.au/english/resources/pedro-scale/ (access for this review: April 14, 2023).

### Statistical analyses

Means and standard deviations of changes from baseline were calculated for each study since there were baseline differences in some of the included studies (Higgins et al., 2019). The mean changes in BALP, CTX and BMD were calculated by subtracting the mean score after the intervention from the mean score before the intervention, whereas the standard deviation of the change was calculated by the equation (Higgins et al., 2019) (correlation coefficient, Corr = 0.5):

$${SD}_{change}=\sqrt{{{SD}_{pre}}^{2}+{{SD}_{post}}^{2}-\left(2\times Corr\times {SD}_{pre}\times {SD}_{post}\right)}$$

Means and standard deviations of changes from baseline and sample size were applied for meta-analysis by RevMan (Review Manager Version 5.4). Effect sizes were set at < 0.2 = trivial, 0.2–0.5 = small, 0.5–0.8 = moderate, and >0.8 = large (Cohen, 2013). All meta-analyses were conducted with a random effects model to determine heterogeneity (*I* ^*2*^) among the studies, and larger than 60% was considered substantial (Schünemann et al., 2013). *p* < 0.05 was considered as the threshold for statistics.

In total, six meta-analyses were conducted. First of all, the effects of LI-BFR training on BALP, CTX and BMD were compared with LI training without BFR (1‒3 analyses). Then the effects of LI-BFR training on BALP, CTX and BMD were compared with HI training without BFR (4‒6 analyses). Additionally, subgroup analyses by different populations (i.e., young adults and old adults) were performed to compare the effects of LI-BFR training on bone metabolism with those of LI training or HI training without BFR.

## Results

### Study selection, characteristics

In total, from an initial 285 screened studies, 12 studies were included in this review (Figure 1). The included studies involved 378 participants with heterogeneous samples (i.e., young adults, old adults and patients undergoing ACLR). The interventions of included studies were based on resistance and aerobic training and varied in duration from 3 to 12 weeks with the frequency of 2‒3 times per week. The load range of HI training was 60%–80% 1RM, and the load range of LI training is 20%–30% 1RM. The BFR cuff pressure was generally 110–220 mmHg or 50%–80% arterial limb occlusion. The specific characteristics of the studies are summarized in Table 1. Among the included studies, all studies achieved 5–7 points (medium-high quality). The PEDro scale score had a median of 6 of 10 points across studies (Table 2).

### LI-BFR *versus* LI resistance training

**FIGURE 2:**
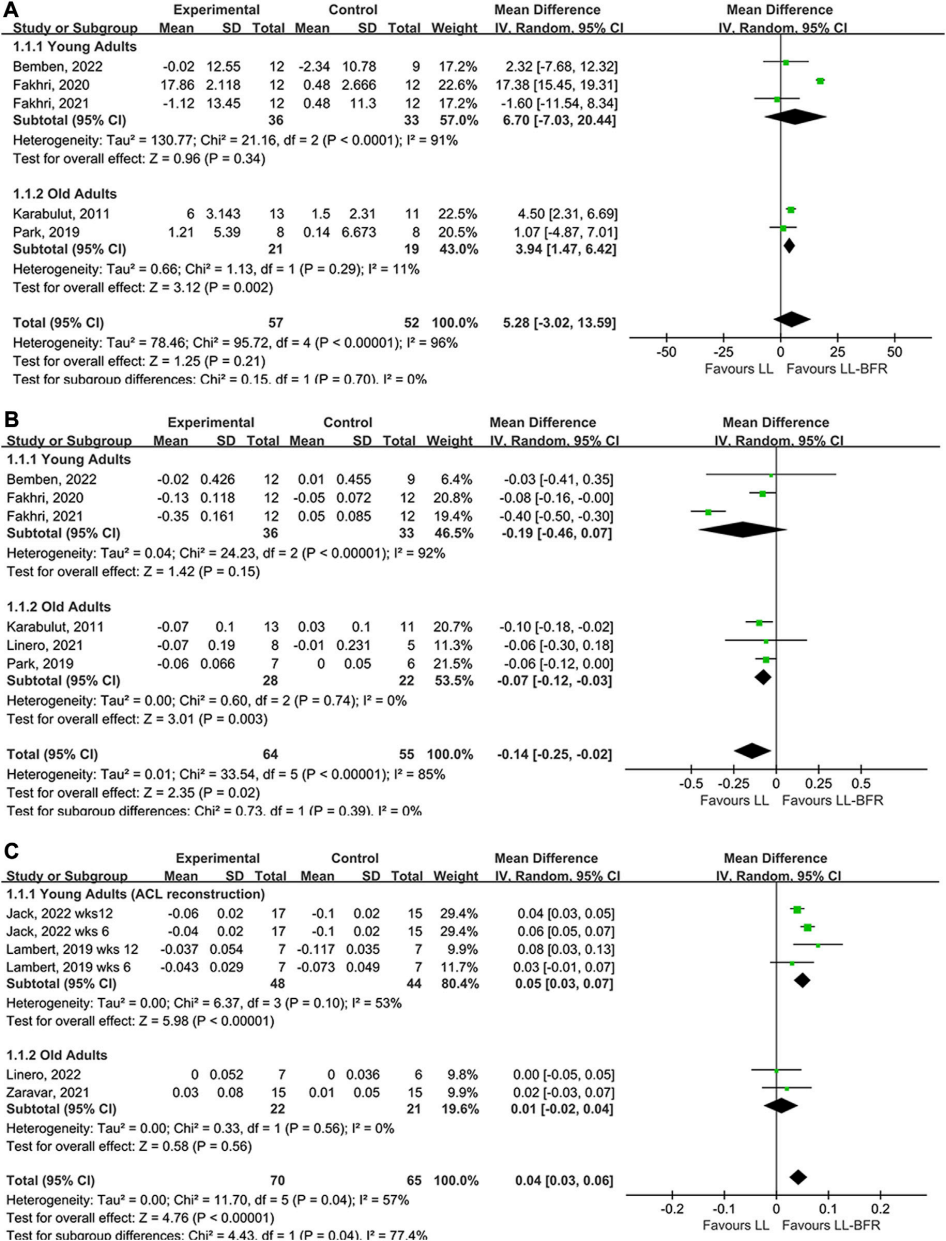
Forest plot illustrating the comparison of LI-BFR to LI training. **(A)**, effects of training on BALP; **(B)**, effects of training on CTX; **(C)**, effects of training on BMD.

The meta-analysis compared the effects of LI-BFR with LI training on BALP (5 studies), CTX (6 studies) and BMD (4 studies). The results of the forest map using a random effect model, which indicated that LI-BFR training had a better impact on bone health than LI training without BFR. Regarding bone turnover makers, LI-BFR training associated with BALP (young adults: MD = 6.70, *p* < 0.00001; old adults: MD = 3.94, *p* = 0.002) had a larger effect than LI training (Figure 2A), and LI-BFR training associated with CTX (young adults: MD = −0.19, *p* = 0.15; old adults: MD = −0.07, *p* = 0.003) showed greater decrements than LI training but not significant for young adults (Figure 2B). Regarding bone content, LI-BFR training associated with BMD (young adults: MD = 0.05, *p* < 0.00001; old adults: MD = 0.01, *p* = 0.47) had a slightly larger effect than LI training but not significant for old adults (Figure 2C).

### LI-BFR *versus* HI resistance training

**FIGURE 3:**
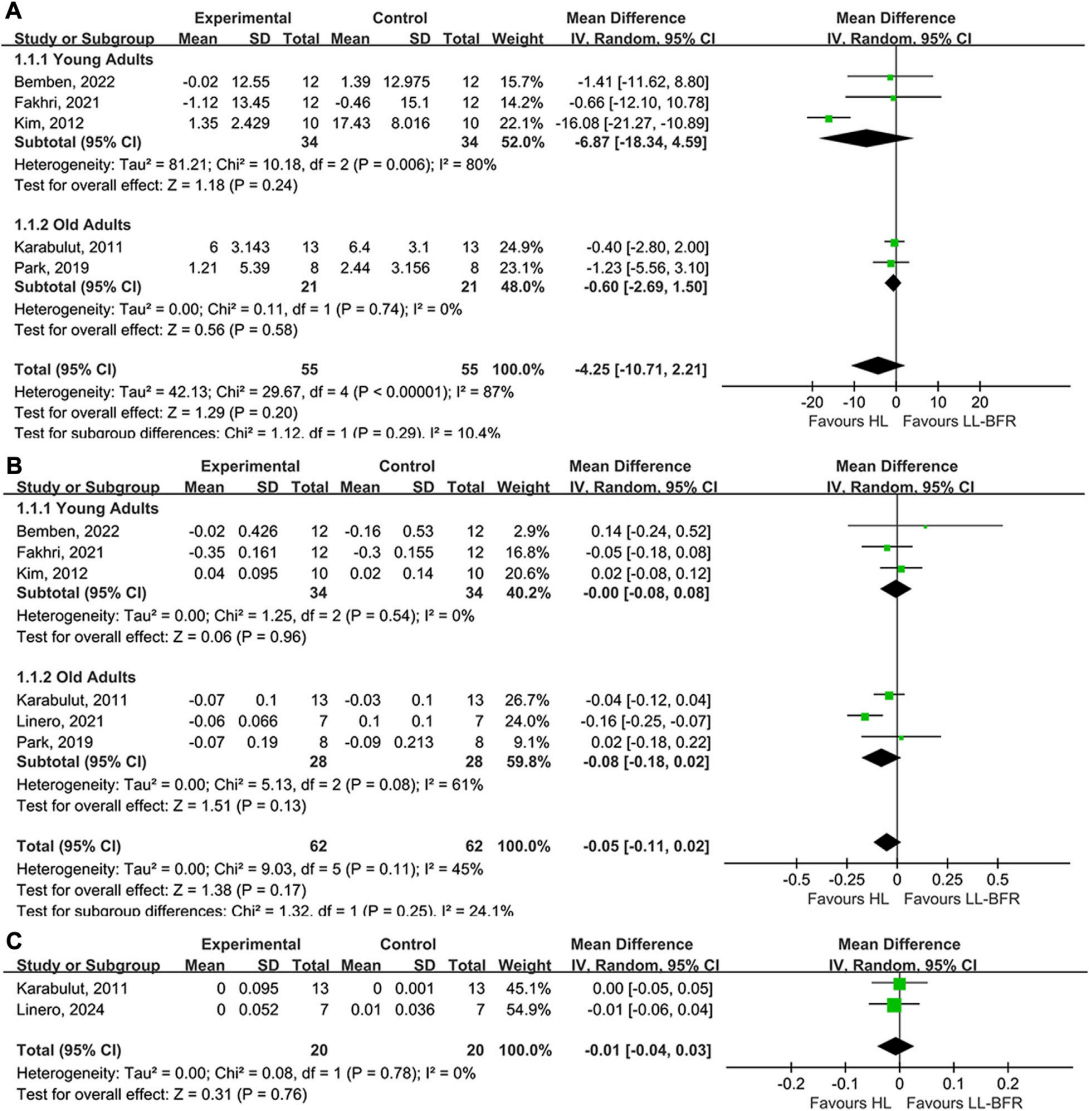
Forest plot illustrating the comparison of LI-BFR to HI training. **(A)**, effects of training on BALP; **(B)**, effects of training on CTX; **(C)**, effects of training on BMD.

The meta-analysis compared the effects of LI-BFR with HI training on BALP (5 studies), CTX (6 studies) and BMD (2 studies). The results of the forest map using a random effect model, which indicated that the effects of LL-BFR training on bone adaption were less effective than that of HI training. Regarding bone turnover makers, LI-BFR training associated with BALP (young adults: MD = −6.87, *p* = 0.24; old adults: MD = −0.6, *p* = 0.58) had smaller increments than HI training but not significant (Figure 3A), and LI-BFR training associated with CTX (young adults: MD = 0, *p* = 0.96; old adults: MD = −0.08, *p* = 0.13) showed a slightly smaller effect than HI training but not significant (Figure 3B). Regarding the BMD, LI-BFR training had a similar effect with HI training (MD = −0.01, *p* = 0.76) but not significant (Figure 3C).

### BFR and walking

**FIGURE 4:**
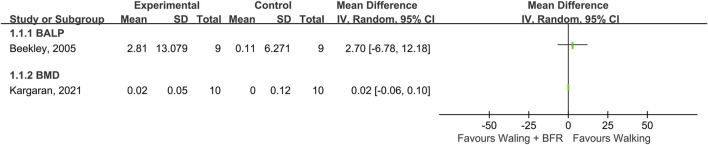
The effects of walking with BFR *versus* normal walking on BALP and BMD.

Two studies investigated the effects of walking training with BFR on bone metabolism compared with walking training without BFR (Figure 4). The study by Beekley et al. (2005) showed that walking training with BFR had a greater impact on BALP increment than walking training without BFR (effect size = 2.7). Another study by Kargaran and Amani-Shalamzari (2022) demonstrated walking training with BFR had a similar effect with walking training without BFR (effect size = 0.02).

## Discussion

To the best of our knowledge, this is the first meta-analysis review that examined the effects of LI-BFR training on bone metabolism when compared to those of LI training (20%–30% 1RM) and HI training (60%–80% 1RM) without BFR. The main finding was that LI-BFR training induced greater improvements in bone health than LI training, but less effective than HI training. Additionally, walking training with BFR had a better effect on bone health than walking training without BFR. This finding underlines the potential of LI-BFR training as an effective and efficient alternative to HI training for promoting gains in bone health in those who are not physically capable of withstanding high mechanical loads, such as untrained individuals, older adults, or patients undergoing musculoskeletal rehabilitation.

Our results suggested that LI-BFR training had a greater increment on bone formation markers (BALP) and a greater decrement effect on CTX than LI training. There are several possible explanations for the differences. Firstly, LI-BFR training could induce additional fluid pressure (i.e., intramedullary pressures and interstitial fluids) due to increased vascular restriction compared to LI training (Loenneke et al., 2012), which may cause a more intense biochemical response of osteocyte membrane or cell (Fritton and Weinbaum, 2009). Secondly, the hypoxic environment induced by BFR could activate hypoxia-inducible transcription factor (HIF) and increase the expression of vascular endothelial growth factor (VEGF), which could further increase the formation of new blood vessels in the bones (Araldi and Schipani, 2010). The transport of bone cell precursors, which are important for osteogenic coupling, is mainly dependent on blood vessels (Lafage-Proust and Roche, 2019). Thirdly, localized ischemia and metabolic stress could lead to adaptive responses that modulate inflammation by regulating inflammatory cytokines (e.g., interleukin-6, endothelin-1and tumor necrosis factor-alpha), thereby inhibiting bone resorption (Rossi et al., 2018). Fourthly, hypoxia and small reductions in pH induced by BFR could promote bone formation and inhibit bone resorption by regulating the secretion of hormones (e.g., insulin-like growth factor 1—IGF-1, growth hormone—GH and parathyroid hormone—PTH) (McCarthy, 2006; Hamrick, 2011). In this review, several studies confirmed that BFR training could better maintain bone health by regulating cytokines and hormones. The study by Park et al. (2019) demonstrated that BFR training had a significant increase in VEGF, that is, related to the formation of new blood vessels in the bones compared with HI and LI training. Three other studies by Beekley et al. (2005), Bemben et al. (2022), Zaravar et al. (2021) found that BFR training had more significant increases in IGF-1, GH and testosterone, which is beneficial to bone formation; and had more significant decreases in PTH, which can inhibit the bone resorption. These advantages of LI-BFR training over LI training are very important for older adults and those undergoing musculoskeletal rehabilitation who are not physically capable of withstanding high mechanical loads. They can achieve better training effects and maintain bone health with low load. Even walking training with BFR can also achieve similar effects on bone metabolism (Beekley et al., 2005; Kargaran and Amani-Shalamzari, 2022). Although LI-BFR training had less impact on bone metabolism than HI training according to the findings of this review, LI-BFR training may be the optimal choice for maintaining bone health in these populations at present. For physically active people and athletes, HI training may be the optimal choice for maintaining bone health.

Interestingly, according to our findings, there was little difference in the effects of LI-BFR training, LI training and HI training on BMD. The most probable reason was that the training intervention period of the included studies was shorter than the normal bone remodeling cycle. Bone remodeling consists of resorption phase (around 2 weeks), reversal phase (around 5 weeks), and formation phase (4 months), which takes approximately 6 months (Eriksen et al., 1984; Eriksen et al., 1984; Agerbaek et al., 1991). The intervention studies included in this review ranged from 3 to 12 weeks, and this period coincides with the reversal phrase and the beginning of the bone formation phrase. Bone formation is a long process lasting about 4 months, and even the 12-week intervention only reached the beginning phase of bone formation. Therefore, short-term intervention may not cause significant changes in BMD. Future studies need to focus on long-term intervention periods (≥6 months) to verify the impact of different training methods on BMD.

Different BFR methods (e.g., cuff pressure, cuff width and BFR type) result in different hemodynamics (Neto et al., 2017a; Zhang et al., 2022), which may have different effects on bone adaptations. Gapper et al. (2021) found that there was no difference in the acute effects of LI-BFR training with 110 mmHg pressure *versus* LI training on bone metabolism among young adults. The most likely reason is that the cuff pressure of 110 mmHg may not be sufficient to elicit any osteocytic response. Conversely, set pressures exceeding the complete arterial occlusion of individuals may result in sports injuries. Therefore, future studies should consider setting cuff pressure according to an individual’s percentage of arterial occlusion pressure (AOP) to mitigate individual differences and ensure training safety. Wide cuffs could reach cuff set pressure at a lower value than narrow cuffs (Jessee et al., 2016). This means that wide cuffs could induce a greater flow restriction than narrow cuffs at the same cuff set pressure, which may result in better effectiveness of BFR training. However, the effects of different cuff widths on bone response could not be observed due to similar cuff widths (5‒7 cm) in this review. Future studies need to explore the effects of BFR training with different cuff pressures and cuff types on bone metabolism. Additionally, the resistance exercise with continuous and intermittent BFR had different effects on hemodynamics (Vilaça-Alves et al., 2016; Neto et al., 2017b). Cuff deflation during rest phases of intermittent BFR could result in an increased venous return, thereby reducing blood lactate concentration (Suga et al., 2012; Okita et al., 2019). As the pH neutralizes, the secretion of cytokines and hormones that facilitate bone adaptation may be reduced. However, the comparative effects of continuous and intermittent BFR on bone metabolism could not be meta-analyzed in this review, because the LI-BFR training in the included studies all used intermittent BFR intervention except for one continuous BFR intervention. Therefore, more studies are expected to consider the effects of BFR type or duration of BFR on bone metabolism. In addition to BFR training interventions, diet and nutrition also play an important role in bone health, especially calcium, vitamin D, and vitamin K_1_ in the diet (Cashman, 2007). Therefore, BFR training combined with diet and nutrition intervention may be a more effective way to prevent osteoporosis and treat bone diseases. Future studies should also focus on the effects of the combined method on bone metabolism.

Despite concerns about hemodynamic derangement and ischemia-reperfusion injury (Fitzgibbons et al., 2012; Waclawovsky and Lehnen, 2016), many reviews on BFR training confirmed that proper implementation had no greater risk than traditional exercise modes (Manini and Clark, 2009; Loenneke et al., 2011; Pope et al., 2013). This review also confirmed this and no studies in this review reported the presence or absence of adverse events. Although injury from BFR training seems rare (Scott et al., 2015), the risk of injury may be exacerbated in clinical populations, such as patients with musculoskeletal rehabilitation or older adults with cardiovascular disease. Therefore, it is important for practitioners to conduct screening and health assessments before BFR training to avoid unnecessary injuries. A clinical screening tool has been developed by Kacin et al. (2015), that is, used to determine risk when prescribing BFR training, including assessments of personal, medical, social and family histories. This tool is important for reducing the risk of injuries caused by BFR training. In addition, the individualization of occlusive pressure is also a training safety issue worth considering, because the same pressure setting may not restrict blood flow to the same degree for different individuals. For example, individuals with larger thigh circumference require greater pressure to reach the same level of occlusion as individuals with smaller thigh circumference (Heitkamp, 2015). This may lead to adverse cardiovascular problems, especially when the set pressures result in complete arterial occlusion. A recent technique for calculating the total arterial occlusion pressure (AOP) has recently emerged, which allows the pressure to be selected at a percentage of individual AOP (AORN Recommended Practices Committee, 2007). This method can avoid the safety issues of complete arterial occlusion and the issue of differences in the degree of blood flow restriction caused by individual differences.

Limitations and prospects of the present meta-analysis review include: 1) There are only a small number of articles meeting the inclusion criteria and limited data on different populations in this review. More studies are expected to further expand the results of this meta-analysis and provide more theoretical support for BFR intervention in bone in the future. 2) This study did not take into account the impact of gender differences, because the number of included studies targeting young adults and older adults groups was too small for moderation analysis. In fact, women are more likely to develop osteoporosis due to differences in peak bone mass and hormone secretion between men and women (Osteoporosis, 2022). These differences in bone adaptation are likely to contribute to gender differences in the effects of BFR training on bone metabolism. Therefore, future research should consider the impact of gender differences in BFR training. 3) This study did not explore the impact of BFR strategy in depth due to the lack of BFR strategy information (e.g., BFR duration, cuff pressure and cuff width) in some included studies and the limitation of research data. More studies are expected to further explore the effects of different BFR strategies on bone metabolism.

## Conclusion

The present meta-analysis shows that LI-BFR training induces greater improvements in bone health (increments in BMD and bone formation, decrements in bone resorption) than LI training, but was less effective than HI training. Additionally, walking training with BFR had a better effect on bone metabolism than training without BFR. Therefore, LI-BFR training may be an effective and efficient way to improve bone health for untrained individuals, older adults, or those undergoing musculoskeletal rehabilitation, and show the positive effect in the prevention and treatment of bone diseases.

## Data Availability

The original contributions presented in the study are included in the article/Supplementary Materials, further inquiries can be directed to the corresponding authors.
